# Nutcracker Syndrome: laparoscopic external stenting of the renal vein (“the shield technique”)

**DOI:** 10.1590/S1677-5538.IBJU.2015.0666

**Published:** 2017

**Authors:** Fernando Korkes, Marcel Silveira, Oseas Castro Neves-Neto, Luiz Franco Brandão, Marcos Tobias-Machado, Nelson Wolosker, Felipe Nasser, Alexandre Maurano

**Affiliations:** 1 Serviço de Urologia, Hospital Israelita Albert Einstein, SP, Brasil;; 2 Departamento de Urologia, Faculdade de Medicina ABC, SP, Brasil;; 3 Serviço de Cirurgia Vascular, Hospital Israelita Albert Einstein, SP, Brasil;; 4 Departamento de Radiologia Intervencionista, Hospital Israelita Albert Einstein, SP, Brasil;; 5 Departamento de Radiologia, Hospital Israelita Albert Einstein, SP, Brasil

## Abstract

Nutcracker syndrome refers to the complex of clinical symptoms caused by the compression of the left renal vein (LRV) between the abdominal aorta and the superior mesenteric artery, leading to stenosis of the aortomesenteric portion of the LRV and dilatation of the distal portion. Hematuria, proteinuria, flank pain, varicocele and pelvic congestion may occur, occurring more frequently in young adults. Conservative management, might be the option whenever it is possible. When surgical treatment is required, classically open surgery have been performed, with major surgeries as LRV transposition or bypass techniques. The main caveats regards the fact that these are large and risky surgeries. Endovascular surgery with venous stent placement has gained some space as it is minimally invasive alternative. However, venous stents are associated with a high number of trombotic complications and in many cases requirement of life-long anticoagulants. External stenting of the LRV with this “shield technique” is a minimally invasive alternative, with good medium term results. We herein demonstrate our second experience with the technique of this surgery in a patient with 12 months of follow up and excellent results.

**Available at: http://www.intbrazjurol.com.br/video-section/korkes_373_373**

